# Western Bluebirds (*Sialia mexicana*) Use Public Information for Prospecting but Not Ultimate Nest Site Selection

**DOI:** 10.1002/ece3.74210

**Published:** 2026-08-18

**Authors:** Fatime W. Jomaa, Cody Pham, Eleanor K. MacDonald, Daniel S. Karp, Matthew D. Johnson

**Affiliations:** ^1^ Department of Wildlife Cal Poly Humboldt Arcata California USA; ^2^ Department of Wildlife, Fish, and Conservation Biology University of California, Davis Davis California USA

**Keywords:** habitat selection, nest box selection, post‐breeding prospecting, public information, *Sialia mexicana*, vineyard

## Abstract

Public information about conspecific presence and reproductive success, in addition to direct environmental sampling, may allow individuals to efficiently assess breeding site quality. We tested the hypothesis that prospecting Western Bluebirds ( *Sialia mexicana* ) use post‐breeding public information to select future nest sites in agricultural landscapes. We further examined effects of breeder age and local habitat characteristics to evaluate whether individual experience and environmental context affected site choice/response to public information, and whether these variables influenced reproductive success. We installed 200 nest boxes across 10 vineyards in Napa Valley, California USA concurrent with final broods of the bluebird breeding season. Boxes in five vineyards were assigned an experimental treatment simulating conspecific presence and reproductive success (old nesting material and playback of adult and nestling vocalizations) or near‐treatment (within auditory range of playback), while boxes in five additional vineyards served as unmanipulated controls. Prospecting activity was quantified using video monitoring. Nest box selection, reproductive success, and local habitat features were assessed the following breeding season. Prospecting activity was 8.47 times higher at treatment boxes and 2.77 times higher at near‐treatment boxes relative to control boxes. This response was mediated by local habitat features: visitation was higher at boxes closer to sparse tree patches and treatment effects declined with increasing nearby tree cover. In contrast, nest box occupancy showed no effect of playback treatment and had a weakly supported but consistent association with similar habitat variables. Breeder age also did not influence settlement responses to public information, and reproductive success was driven primarily by clutch initiation date rather than age/habitat features. Our results suggest that public information may function as a strong post‐breeding recruitment cue but do not directly determine nest site selection in this resident songbird population, while final settlement may rely more heavily on direct habitat assessment.

## Graphical abstract

Prospecting Western Bluebirds demonstrated a strong response to public information simulating reproductive success within nest boxes deployed in agricultural systems, but this initial attraction did not translate to increased box selection the following breeding season. Instead, settlement was driven primarily by local habitat features involving woody vegetation, indicating that Western Bluebirds may use public information to locate potential sites but rely on direct environmental assessment when choosing where to breed. Our findings inform strategies for improving nest box placement in agricultural systems, and clarify how public information and local habitat structure may interact to shape settlement decisions in a resident songbird species.

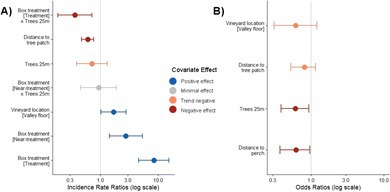

## 1 Introduction

Habitat selection has important consequences for avian fitness and population dynamics (Johnson and Wood 2018). When selecting breeding sites, individuals often explore and evaluate multiple locations, a behavior known as prospecting (Reed et al. 1999). Poor nest site choice can reduce reproductive success through lowered parental investment or offspring survival (Williams 1966; Charnov and Krebs 1974), favoring the evolution of mechanisms that allow individuals to assess habitat quality accurately (Cody 1981). Prospecting birds thus commonly rely on environmental cues such as food abundance and vegetation structure to guide these decisions (Winter et al. 2005; Crampton et al. 2011). However, direct sampling of unfamiliar habitat can be energetically costly, such as for inexperienced natal dispersers or migrating individuals with limited time resources.

In addition to personal information gained through direct assessment, prospecting birds may use inadvertent social information (ISI), or secondhand information obtained by observing conspecific presence or performance (Danchin et al. 2004). ISI includes *location cues*, in which the presence of conspecifics or individuals with similar resource needs serves as an indicator of where resources may be found, and *public information*, which provides insight on resource quality based on individual performance, such as evidence of successful breeding (Valone and Templeton 2002; Danchin et al. 2004). Public information is thought to be especially valuable during post‐breeding prospecting, when reproductive outcomes are visible to individuals evaluating future breeding sites (Boulinier et al. 1996; Pärt et al. 2011).

Experimental manipulations of ISI have shown that simulated conspecific presence in the form of playback and/or decoys can attract prospecting birds in both colonial and non‐colonial migratory avian species (Dunlop et al. 1991; Podolsky and Kress 1992; Jeffries and Brunton 2001; Ward and Schlossberg 2004; Betts et al. 2008; Kelly and Schmidt 2017). Manipulations mimicking reproductive success, such as increased brood size and provisioning, further demonstrate that birds may use breeding performance‐based cues when evaluating nest sites (Pärt and Doligez 2003; Doligez et al. 2004). Responses to ISI, however, can vary among individuals and species. Younger or less experienced birds may rely more heavily on social cues than older, experienced breeders, likely due to limited opportunities for personal sampling (Nocera et al. 2006; Ahlering et al. 2010). Furthermore, most ISI studies focus on migratory species and demonstrate an overall positive influence of social cues on settlement decisions, but limited research exists regarding these effects on resident populations (Ahlering et al. 2010; Valente et al. 2021).

Understanding how different species and individuals with varying levels of breeding experience utilize ISI, and the degree to which they integrate it with direct assessment of habitat features, is particularly relevant in human‐modified landscapes. In agricultural systems, natural nesting opportunities are often limited, and nest boxes are thus often used to attract insectivorous, cavity‐nesting birds that may contribute to pest control (Kross and Baldwin 2016; Jedlicka et al. 2017; Bouvier et al. 2022). Occupancy rates in artificial nest boxes, however, can be site‐dependent, suggesting that nest site selection depends on more than box availability alone: landscape context, vegetation structure, and proximity to natural habitat may all influence box colonization and persistence across species (Navara and Anderson 2011; Brown et al. 2014; Wendt and Johnson 2017).

We examined a resident population of Western Bluebirds ( *Sialia mexicana* ) in Napa Valley, California USA. Western Bluebirds in established interior populations away from range edges are generally philopatric and often acquire territories close to their natal sites (Duckworth 2008). They are obligate secondary cavity nesters that are historically associated with oak woodland habitats in California (Purcell 2011). In part because oak woodlands have declined due to agricultural expansion (Mummert et al. 2002), Western Bluebirds are now identified as an at‐risk oak‐associated species by the North American Bird Conservation Initiative (NABCI 2025). Although nest boxes on farms can partially compensate for the loss of natural cavities, occupancy remains spatially variable. Surveys of 1007 nest boxes across 17 vineyards in Napa Valley found that fewer than one‐third were occupied by Western Bluebirds, with 49% remaining empty (Martinico et al. unpublished data). While this variability suggests that local habitat features could play a role in settlement decisions, social cues may also shape nest box use. Because Western Bluebirds typically lay multiple broods within a breeding cycle (Keyser et al. 2004), ample opportunity exists for individuals to observe the reproductive outcomes of earlier nests within a single season. Notably, such post‐breeding extraterritorial visits by Western Bluebirds have been linked to increased box occupancy in subsequent years (Smith et al. 2020), pointing to a potential role for public information in nest site assessment. Despite growing interest in deploying nest boxes to attract pest‐eating birds in agricultural habitats, the influence of public information on avian nest site selection within agroecosystems, and for Western Bluebirds specifically, remains largely untested.

The aim of this study was to test the hypothesis that prospecting Western Bluebirds use public information, in combination with direct assessment of local habitat features, during the post‐breeding period to inform nest site selection the following year. Specifically, we evaluated whether prospecting activity and subsequent nest box occupancy were influenced by (i) experimentally supplied public information simulating conspecific presence and reproductive success, (ii) local habitat features, and (iii) breeder age, as a proxy for experience. We predicted that (1) prospecting activity would be higher at experimental boxes broadcasting public information compared to control boxes, (2) nest box occupancy the following breeding season would be greater at experimental than control boxes, and (3) preferential Western Bluebird habitat features such as open land and sparse woody vegetation would still mediate responses to public information for a resident population with greater opportunity to sample sites directly. We further predicted that (4) second‐year birds (SY; individuals entering their first breeding season) would show stronger responses to public information than after‐second‐year birds (ASY; individuals with one or more past breeding opportunities), and that (5) SYs would have lower reproductive success compared to ASYs if they prioritized simulated public information over personal information during nest site selection, as reproductive success is expected to vary with the suitability of local habitat features.

## 2 Methods

### 2.1 Study Area

**FIGURE 1:**
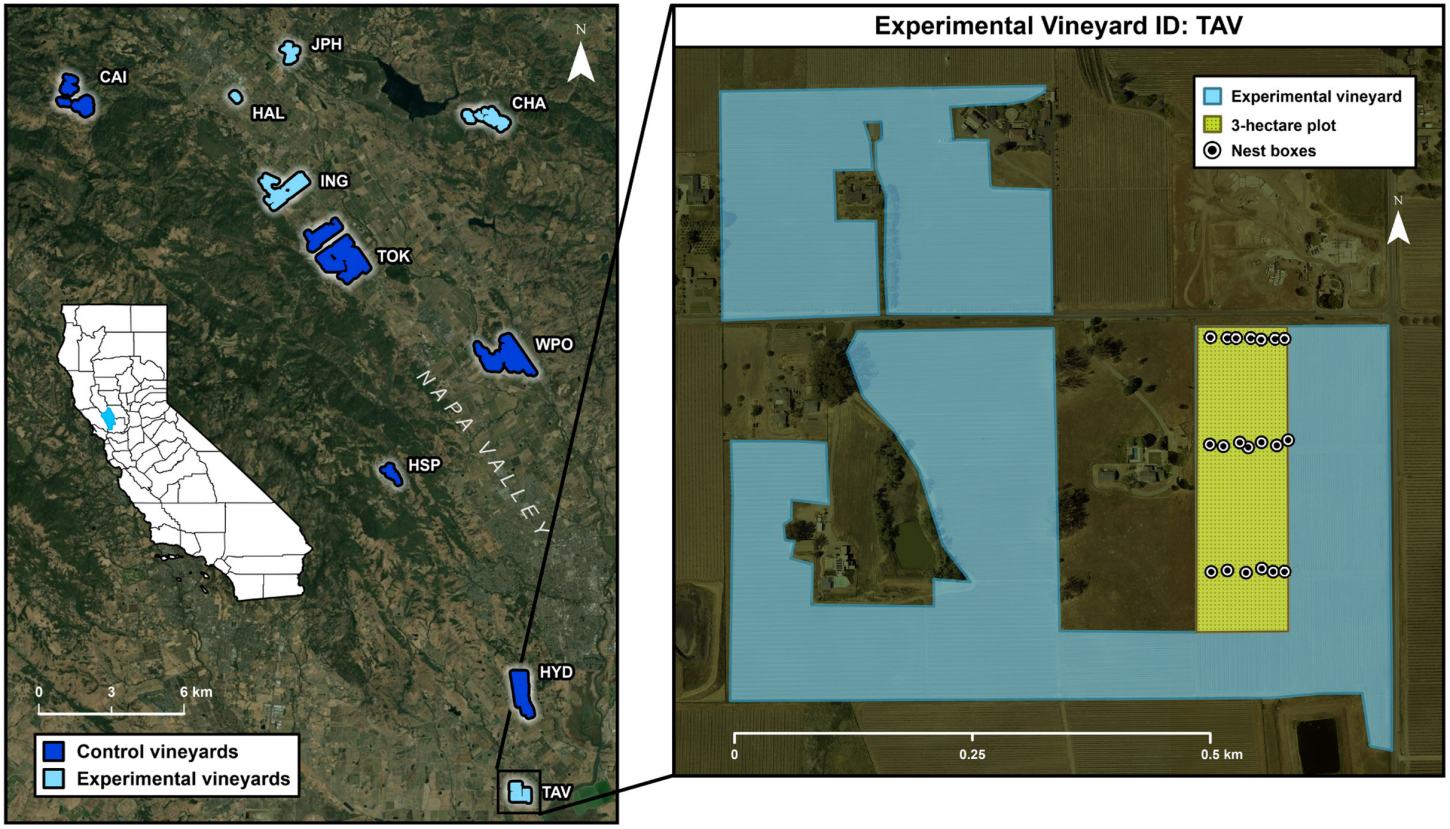
(Left) Napa Valley study area with control (dark blue) and experimental (light blue) sites highlighted; (right) 3‐ha nest box plot highlighted in yellow within an experimental site (TAV). Nest boxes were installed at the ends of vine rows.

The study was conducted in Napa Valley, California, USA (Figure 1), a region characterized by a dry Mediterranean climate. Across focal vineyards, mean annual temperatures averaged 15.38°C (range: 12.33°C–17.05°C) and annual rainfall averaged 830.6 mm (range: 141.5–1662.5 mm) between 2000 and 2024 (Thornton et al. 2020). The region supports ~18,400 ha of vineyards within the valley floor and foothills (Gaiser 2025) and hosts a diverse mosaic of habitats primarily along the valley edges, with mixed woodlands, oak savannah, and grasslands in the south and mixed oak scrub and conifer forests in the north (Napa County 2010).

Ten vineyards (Figure 1) were selected to represent variation in local (within‐farm) habitat complexity and landscape composition. Six sites were located near the valley floor, in simplified, agriculture‐dominated landscapes, and four occurred along valley edges or in the adjacent hills with higher densities of natural habitats nearby. Experimental and control vineyards were distributed evenly across these landscape types to avoid confounding treatment with habitat context. Elevations spanned 14–613 m. All vineyards shared similar viticultural practices (i.e., no insecticides, periodic fungicide applications, routine vine pruning). Although the vineyards differed in overall property size, all study plots consisted of three‐hectare areas within each vineyard, and surrounding land outside the vineyard borders was similarly utilized by neighboring vineyard properties. Companion point count surveys confirmed that Western Bluebirds were distributed across the study region without any underlying north–south gradient in local abundance.

### 2.2 Post‐Breeding Prospecting Protocol

Vineyards had few pre‐existing songbird nest boxes (*n* = 14 boxes at two of the 10 sites). Given their low abundance relative to the vineyards' large spatial extents, any effects of existing boxes were expected to be minimal. In late June 2023, we installed nest boxes (Audubon Red Cedar Bird House) on ~3 ha plots (1/2″ Conduit/Rebar Pole Mounting System 2012) at each of 10 vineyards (200 boxes total). Installation was timed to coincide with Western Bluebird second broods, when adults were still actively breeding but expected to begin post‐breeding prospecting.

Contingent on the configuration of the vineyard block, we usually placed boxes at the ends of vine rows to minimize disruptions to vineyard operations, consistent with standard practice across the region (Figure 1). Specifically, at each vineyard, we placed 20 boxes within ~3 ha plots, spaced between 10 and 35 m apart from their nearest neighbor. Based on average bluebird territory sizes in California (~1.3 ha; Kraaijeveld and Dickinson 2001), nest box spacing is often recommended to be larger. That said, our intent was to mimic standard practices in our study region, where growers regularly place nest boxes in close proximity and at high densities to bolster biological pest control. For example, among 267 nest boxes occupied by bluebirds across our study region in a companion study (Martinico et al. unpublished data), neighboring bluebirds were recorded as close as 4 m, and the lower quartile nearest neighbor distance was only 26 m. Thus, we suspect our close spacing did not strongly deter bluebirds. Nevertheless, the same companion study did find some evidence of conspecific nest avoidance, with bluebirds tending to prefer wider spacing (> 50 m), suggesting our high densities could have resulted in slightly lower occupancy rates across all of our experimental treatments.

Within experimental vineyards (*n* = 5), 12 boxes received a treatment consisting of a speaker broadcasting adult and nestling bluebird vocalizations as well as an old bluebird nest collected from the region the previous year placed inside. Avian response to public information may operate at scales larger than an individual nest site (Pärt and Doligez 2003), and given the proximity of all boxes, the whole plot on experimental vineyards experienced some degree of enhanced public information. Therefore, the remaining 8 boxes at each experimental vineyard were categorized as “near‐treatment” boxes. These boxes did not receive a nest or playback speaker, but were located near 1–2 treatment boxes (mean distance: 20 m; range: 8–48 m); every 3rd, 5th, 7th, 9th, 11th, 13th, 15th, and 17th box was designated as near‐treatment. All boxes at the remaining five control vineyards served as untreated controls (*n* = 100).

Playback files were created in Audacity Team 2014 using local nestling recordings collected in 2023 from an active nest box in the study area, as well as songs and calls from adults in California, sourced from Cornell's Macaulay Library (ML111074, ML125391, ML241079311). Specifically, two ~20 s nestling begging sequences were recorded from a 10‐day‐old nest of five nestlings using a Yemenren R9 voice recorder held ~10 cm from the box entrance. We alternated 45 s sequences of begging calls with 90 s sequences of silence (135 s total). Every 5th sequence was followed by 120 s of adult song, 60 s of silence, and 45 s of adult calls. After the 10th begging/silence sequence, the adult sequence was overlapped with nestling begging. This 30‐min loop was then repeated 16 times for an 8‐h playback. Although published calling and begging rates for Western Bluebirds were unavailable, the temporal structure of these sequences was based on a prospecting study in zebra finches (Brandl et al. 2019) and was consistent with calling and begging patterns we routinely observed in the field. Playback was loaded into mp3 players (Dyzeryk MP3) connected to speakers (onn. model # AABLSV100081918) that were then placed directly beneath treatment boxes. Amplitude was calibrated to approximate natural nestling vocalizations; at a 10 m distance, speaker playback produced an Leq of 54.3 dBA. While we were unable to record sound levels from an active nest of five nestlings, an adjacent nest of three 10‐day‐old nestlings produced an Leq of 42.2 dBA at the same distance.

To quantify prospecting, we deployed Akaso Brave 4 cameras on a subset of boxes due to limited video equipment. At experimental sites, all 12 treatment boxes and 6 of 8 near‐treatment boxes were filmed (all near‐treatment boxes except the 7th and 13th). At control sites, cameras were placed on every 2nd, 4th, 8th, 12th, 16th, and 20th box to avoid spatial clustering. Cameras were positioned ~2 m away from boxes and set to record continuously (720P, 30 fps, medium angle). In experimental vineyards, playback was only broadcast during active filming periods. Playbacks and video recordings were conducted between 3 and 19 July 2023, with vineyards recorded in paired experimental‐control bouts to control for confounding weather and temporal variables. Each camera was deployed for 15 h over three consecutive days (0600–1200 on 2 days, and between either 0600–0900 or 1500–1800 on the third). Total video coverage included 60 treatment, 30 near‐treatment, and 30 control boxes.

Footage was processed using the YOLOv10 object detection model from Ultralytics to identify frames containing birds. For each video frame, a Python script ran inference on the model. If a bird was detected, then that frame was marked for output and used to create relevant video clips. We assessed model accuracy by reviewing ~8 h of footage from four videos and comparing recorded activity to video clips produced by the YOLOv10 model for the same four videos. The model captured 100% of desired prospecting events with minimal false positives.

All video clips were then manually reviewed to extract prospecting data. Bluebird prospecting behavior was categorized as follows: (1) within 2 m (proximity to box without direct contact), (2) on box, (3) on entrance hole, and (4) in box. Each discrete action was counted as a separate event, including sequential behaviors or repeated visits. We recorded the sum occurrence of each behavior for each box as well as the overall sum of prospecting activity.

### 2.3 Nest Box Monitoring

Twelve boxes were removed from one experimental site due to unplanned farming operations 6 months after the prospecting experiment but prior to nest box monitoring in the subsequent breeding season. In February 2024, we thus installed 20 additional near‐treatment boxes 100–400 m from the original remaining treatment (*n* = 5) and near‐treatment (*n* = 3) boxes in an adjacent vine block. Total 2024 occupancy monitoring thus included 208 boxes (53 treatment, 55 near‐treatment, 100 control); 110 of these were also camera‐monitored in 2023 (53 treatment, 27 near‐treatment, 30 control). Exclusion of either the additional boxes or the experimental site altogether did not alter overarching trends observed in box use, while inclusion provided stronger support for habitat‐related box selection. Therefore, we retained all boxes in later analysis.

Boxes were monitored twice weekly from April–July 2024 for occupancy and reproduction. Western Bluebirds and Tree Swallows ( *Tachycineta bicolor* ) were the primary occupants, with infrequent nesting by House Finches ( *Haemorhous mexicanus* ), House Wrens ( *Troglodytes aedon* ), and House Sparrows ( *Passer domesticus* ); the latter are non‐native and were thus removed upon discovery. Nesting species were identified by distinct nest and egg morphologies.

Clutch initiation date was recorded for nesting Western Bluebirds. Complete clutches contained between 3 and 6 eggs and were monitored daily from the 12th day of incubation to determine hatch date (day 0), then checked between days 5–12 and 13–16 to track survival. To avoid premature fledging, nests were left undisturbed for 10 days after day 16. Fledging success was determined by the presence of significant fecal matter, while clean nests indicated predation. Nestlings deceased in the nest from unknown causes were recorded.

### 2.4 Adult Capture

Adults were captured opportunistically (*n* = 61 females; *n* = 60 males) when nestlings were 0–16 days old using a Bluetooth‐triggered device (SwitchBot Smart Switch Button Pusher) fit with a cardboard flap and mounted over the box entrance. Capture success was comparable across treatments: we captured 43 of 66 adults nesting in control vineyards (*n* = 28 females; *n* = 25 males) and 68 of 80 adults nesting in experimental vineyards (*n* = 33 females; *n* = 35 males). We aged birds as second‐year (SY) or after‐second‐year (ASY) based on molt limits. All procedures were approved by the Cal Poly Humboldt IACUC (approval number: 2023W12‐A).

### 2.5 Local Habitat Feature Data

Local habitat feature data were collected for each box between April and May 2024. We emphasized local habitat variables (nearby woody vegetation and distance to tree patch) shown previously to negatively affect bluebird nest box use in an extensive regional survey of Napa Valley vineyard boxes (Martinico et al. unpublished data). Specifically, we conducted a visual estimate of the percent woody vegetation (riparian or woodland) within a 25 m radius of the nest box and used a rangefinder to obtain the distance from the nest box to the nearest tree patch (defined as a patch at least 25 m^2^ in area and 4 m in height). In addition, we measured the distance to the nearest possible perch, defined as any structure at least 3 m in height, as Western Bluebirds commonly forage from elevated perches (Herlugson 1983).

## 3 Data Analysis

### 3.1 Data Exploration and Preparation

All analyses were conducted using R (v4.4.2; R Core Team 2024). We created model sets for prospecting activity, box use, and reproductive success (clutch size and fledging success). We scaled and centered all linear predictors, determined that linear forms of local habitat terms outperformed quadratic forms using univariate GLMs and AICc (Akaike 2025), and assessed collinearity using Pearson's correlation coefficients. Although perch structures sometimes coincided with trees, distance to perch was not strongly correlated with tree cover or distance to tree patches (|*r*| < 0.7), so we evaluated prospecting and box use models with and without this variable. Because it showed moderate support in the top box use model, distance to perch was retained as a baseline habitat covariate in subsequent reproductive models. We considered interactions between box treatment (treatment, near‐treatment, and control) and the two key local habitat features identified in the regional survey (distance to tree patch and percent tree cover within 25 m) for prospecting and box use models. For reproductive models, we additionally considered interactions among female age, box treatment, and key local habitat features. Predictors were considered significant if their 95% confidence intervals did not overlap zero. Predictors were identified as trending positively or negatively when their confidence intervals overlapped zero, but their standard error did not.

The experimental design involved treatment assignment at the vineyard level, with individual nest boxes nested within vineyards. Because vineyard‐level treatment (experimental vs. control) did not vary within sites, inference for treatment effects was based on a limited number of independent experimental units, and inclusion of a vineyard‐level random intercept led to convergence issues and unstable variance estimates, likely due to the small number of sites. Therefore, we did not include any random intercepts in subsequent models, instead including vineyard location (foothills vs. valley floor) in all models (except the full null models) to account for variation caused by broader landscape characteristics.

### 3.2 Analysis of Prospecting Activity

We treated total prospecting activity as a discrete response variable. A principal component analysis demonstrated that all four prospecting behaviors (within 2 m, on box, on entrance, in box) loaded positively onto the first principal component, which explained 89.7% of the total variance. Therefore, we generated a composite total prospecting score per box by summing all behavioral events observed over the three‐day filming period. This score was then used as the response variable for subsequent modeling.

Because prospecting data contained excess zeros, we fit zero‐inflated negative binomial models (ZINB) using *glmmTMB* (v1.1.11; Brooks et al. 2017). Count model components explored combinations of local habitat features and box treatment. For the zero‐inflation component, we retained box treatment as the sole predictor because it was the only factor expected to influence whether a box was ever prospectable (i.e., structurally available or attractive) during the three‐day playback window shortly after boxes were installed. The top model was selected via AICc and model weights using *MuMIn* (v1.48.11; Bartoń 2025). Model fit was assessed using DHARMa residual and diagnostic plots (v0.4.7; Hartig 2024). Coefficients were exponentiated to interpret incidence rate ratios (IRR) for count components and odds ratios (OR) for zero‐inflation components.

### 3.3 Analysis of Box Use

We modeled nest box use in 2024 using single‐component binomial GLMs (*lme4* v1.1.37; Bates et al. 2015), with a binary response variable (0 = unused, 1 = used). We fit the same covariate combinations as prospecting models. We also initially evaluated heterospecific occupancy (i.e., boxes used by other species) as a covariate, but it showed no effect on Western Bluebird box use (consistent with our observation that Western Bluebirds on our plots initiated clutches over a month earlier than heterospecifics), and was therefore excluded from the final model set. AICc and model weights were compared using *MuMIn*. Residual and diagnostic plots were evaluated using the *performance* package (v0.13.0; Lüdecke et al. 2021).

A two‐proportion Z‐test was used to determine whether the age structure of bluebirds nesting on experimental vineyards differed from the overall age structure of bluebirds nesting within all study sites. We used the pooled age distribution across all vineyards as an estimate of the population age ratio in the study region. We chose to perform tests for both sexes, as paired bluebirds frequently varied in age between sexes.

### 3.4 Analysis of Reproductive Success

We used package *ordinal* (v2023.12.4.1; Christensen 2019) to conduct ordinal logistic regression to analyze the relationship of clutch size (3–6 eggs) with combinations of box treatment, female age, clutch initiation date, and local habitat features. To perform model validation, we conducted a scale test to determine whether the proportional odds assumption held true for the top model.

Fledging success was calculated as the proportion of nestlings fledged per hatched eggs. We initially fit binomial GLMs and evaluated overdispersion by calculating the dispersion parameter phi (residual deviance divided by residual degrees of freedom). In all models, the dispersion parameter was greater than 1, indicating overdispersion. To accommodate this, we refit the candidate model set using the ‘glm.binomial.disp’ function from the package *dispmod* (v1.2; Scrucca 2018), which estimates overdispersed binomial logit models, with the same covariate combinations as clutch size models. AICc and model weights were assessed with *MuMIn*. Model validation involved manual calculation of deviance residuals. We created (1) a binned residuals versus fitted values plot and (2) binned residuals versus individual predictor plots for all covariates included in the candidate model set.

## 4 Results

### 4.1 Prospecting Activity

The zero‐inflation component of the top model for prospecting by Western Bluebirds in 2023 provided insight into how simulated public information influenced the probability that a box received any prospecting. The zero‐inflation portion predicted the likelihood that an observed zero was a structural zero (a box that was truly never prospected). Compared to control boxes, treatment boxes had 95% lower odds of belonging to the structural zero process, and near‐treatment boxes had 85% lower odds (Table S1). Thus, boxes exposed to playback were far less likely to be boxes that received no prospecting whatsoever, indicating that our simulated public information treatments increased the probability that a box was prospectable.

**FIGURE 2:**
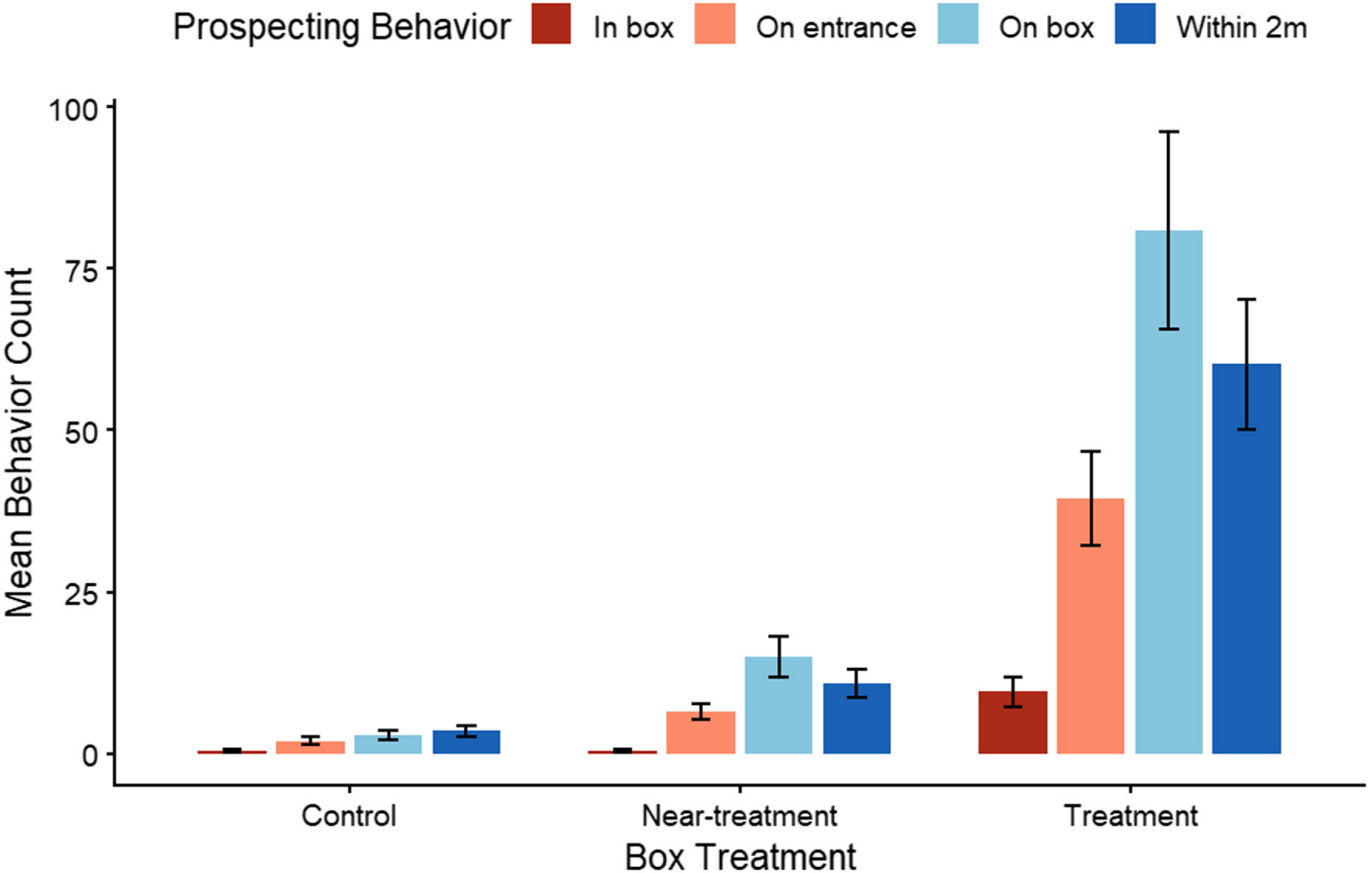
Grouped bar graph displaying mean prospecting behavior counts (within 2 m, on box, on entrance, in box) for each box treatment category (control, near‐treatment, treatment). Black bars represent standard error. Treatment boxes received significantly more prospecting than near‐treatment and control boxes. Near‐treatment boxes received significantly more prospecting than control boxes.

**FIGURE 3:**
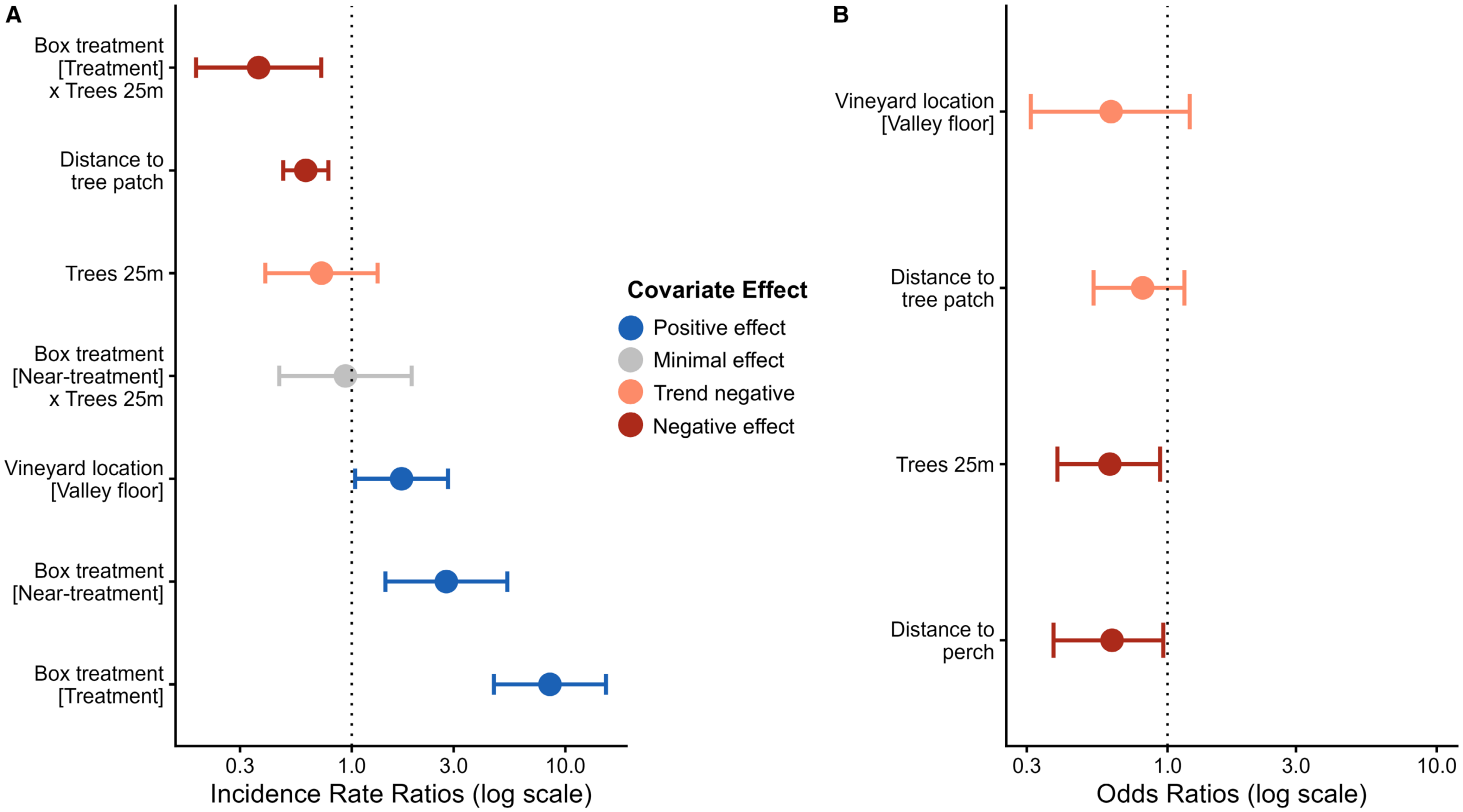
Covariate effects from (A) the prospecting model and (B) the box‐use model. Plot A shows incidence rate ratios (IRRs) from the zero‐inflated prospecting model, representing multiplicative changes in the rate of prospecting activity received by each box. Plot B shows odds ratios (ORs) from the box‐use model, representing changes in the likelihood of Western Bluebird box use. Both IRRs and ORs are plotted on a log scale. Points show estimated effects and bars show 95% confidence intervals. Colors indicate effect direction and strength: Blue/red = significant positive/negative effects (CI and SE exclude 0), orange = negatively trending effects (CI overlaps 0 but SE does not), and gray = minimal effects (both CI and SE include 0).

**FIGURE 4:**
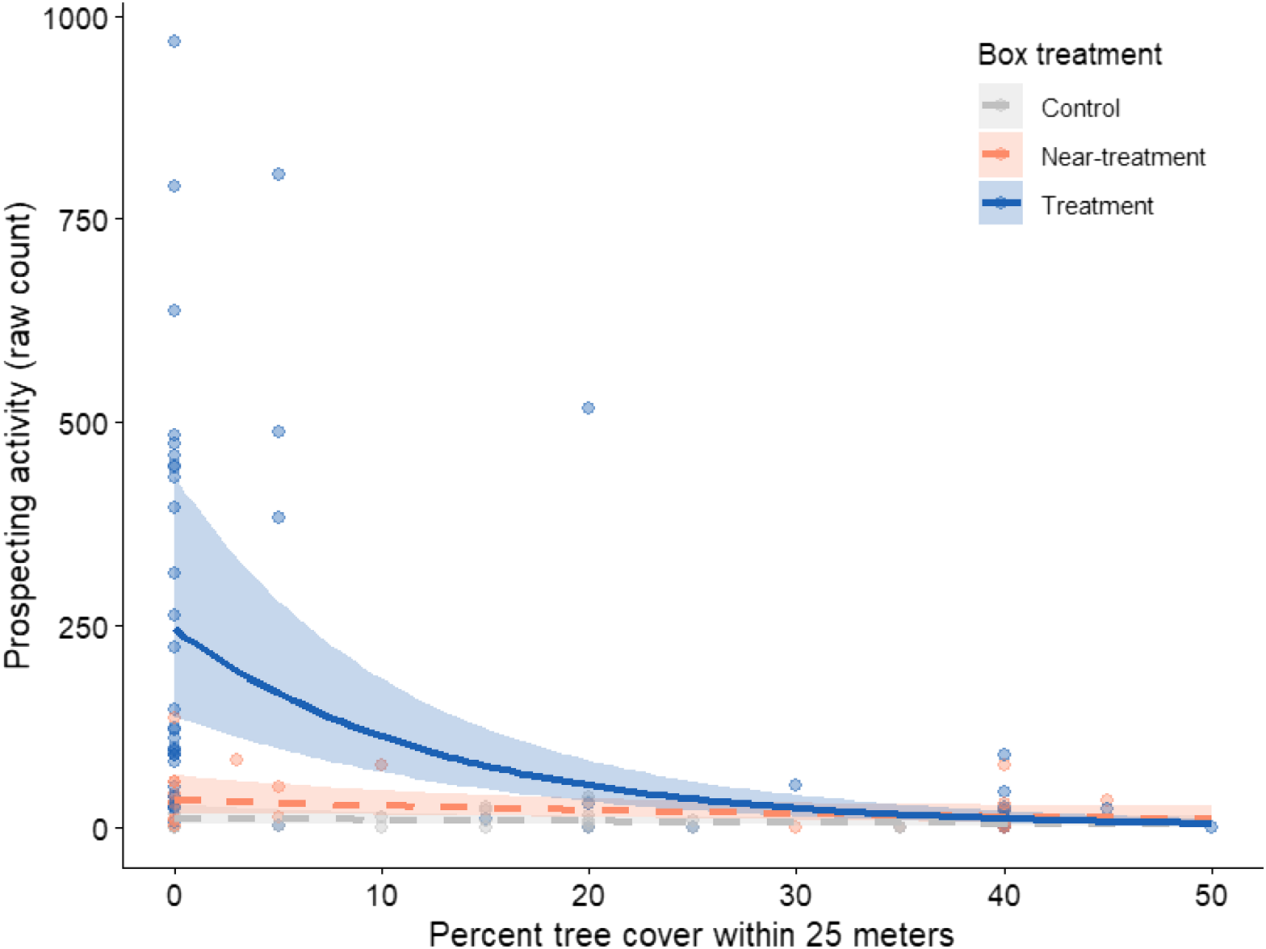
Relationship between percent tree cover within 25 m of a nest box and prospecting activity across box treatment groups. The treatment group (solid blue line) shows a significant interaction with tree cover, with prospecting activity declining as nearby tree cover increases. Near‐treatment and control groups (dashed lines) show no significant interaction with tree cover. Shaded ribbons represent 95% confidence intervals and points reflect raw prospecting activity counts.

Conditional on a box being prospectable, the count component of the model supported the prediction that simulated public information also substantially increased prospecting activity (Figure 2). Relative to control boxes, prospecting activity was 2.77 times higher at near‐treatment boxes and 8.47 times higher at treatment boxes (Table S1; Figure 3A). The model also indicated that local habitat features mediated treatment effect strengths (Table 1). Prospecting activity decreased significantly with increasing distance to the nearest tree patch, but also trended negatively with increasing percentage of trees within a 25 m radius of a nest box (Table S1; Figure 3A). The interaction between box treatment and tree cover within 25 m further revealed that prospecting activity significantly declined with increasing tree cover at treatment boxes, though this effect was not significant for near‐treatment or control boxes (Figure 4). Prospecting activity was also higher at vineyards located on the valley floor, reflecting the greater proportion of experimental vineyards present within that landscape (Table S1; Figure 3A).

**TABLE 1:** Prospecting activity candidate model set.

Model	*k*	AICc	ΔAICc	Weight
**P6: Box treatment × trees 25 m + distance tree patch + Vineyard location**	**12**	**1033.3**	**0**	**0.561**
P5: Box treatment × trees 25 m + distance tree patch + distance perch + Vineyard location	13	1035.5	2.21	0.185
P2: Box treatment × trees 25 m + box treatment × distance tree patch + Vineyard location	14	1036.1	2.81	0.137
P1: Box treatment × trees 25 m + box treatment × distance tree patch + distance perch + Vineyard location	15	1036.5	3.22	0.112
P9: Box treatment + trees 25 m + distance tree patch + Vineyard location	10	1045.0	11.66	0.002
P4: Box treatment × distance tree patch + trees 25 m + Vineyard location	12	1045.6	12.24	0.001
P7: Box treatment + trees 25 m + distance tree patch + distance perch + Vineyard location	11	1047.3	13.96	0.001
P3: Box treatment × distance tree patch + trees 25 m + distance perch + Vineyard location	13	1047.8	14.51	0
P10: trees 25 m + distance tree patch + Vineyard location	8	1068.0	34.73	0
P8: trees 25 m + distance tree patch + distance perch + Vineyard location	9	1070.3	36.94	0
P11: Vineyard location	6	1097.6	64.31	0
P12: Null	5	1110.0	76.67	0

*Note:* Table depicts count model coefficients, parameters (*k*), AICc score, ΔAICc, and model weights. Total prospecting is the response variable for all models. The binomial zero‐inflation portion of the model (not included in table) models the probability of a structural zero and is always predicted by box treatment. P6 (bolded) is the top model with no other models falling within 2 ΔAICc.

### 4.2 Box Use

**FIGURE 5:**
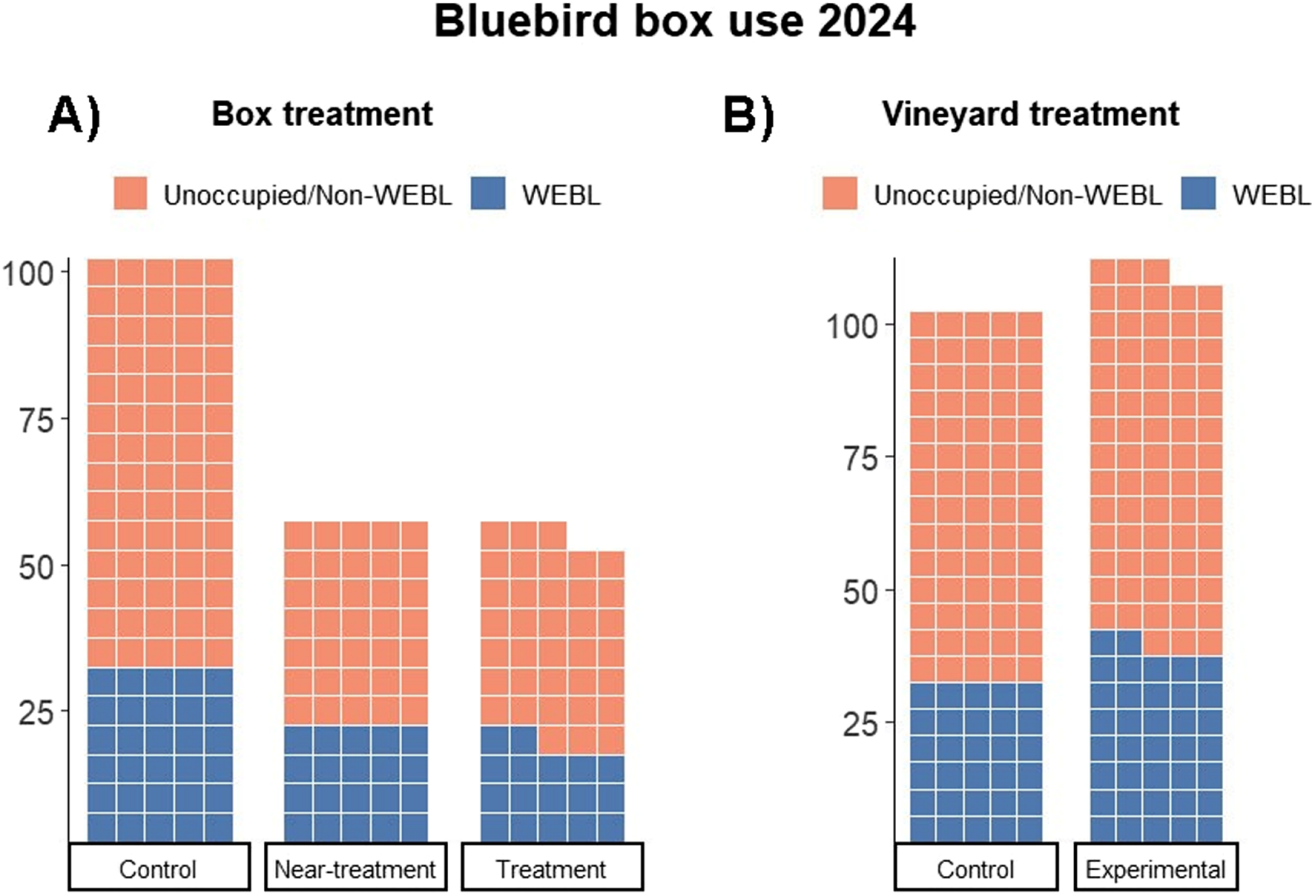
Waffle charts for nest box use in 2024. Each square represents one nest box. Blue squares represent a box selected by Western Bluebirds (WEBL) while orange squares indicate a box left unoccupied or utilized by a different species. (A) Box use broken down at the box treatment level; (B) Box use at the vineyard treatment level.

The top models for box use did not include box treatment, providing little support for the prediction that bluebird used simulated public information to select nest boxes in the following year (Table 2). Indeed, the raw occupancy rate was similar between treatment (32%), near‐treatment (36%), and control (30%) boxes (Figure 5B), as well as at the vineyard scale (experimental = 34%; control = 30%, Figure 5A). Model selection uncertainty was also high; the null model and vineyard location‐only model both fell within 2 ΔAICc of the most heavily weighted model (Table 2). However, local habitat trends that predicted prospecting intensity re‐emerged during box selection in the highest weighted model (Table S1). The probability of box use declined significantly with increasing percent tree cover within 25 m, and showed a negative trend with increasing distance to the nearest tree patch (Table S1; Figure 3B). Box use significantly declined with increasing distance to the nearest perch (Table S1; Figure 3B). A weak negative trend was also associated with vineyards located on the valley floor (Table S1; Figure 3B).

**TABLE 2:** Box use candidate model set.

Model	*k*	AICc	ΔAICc	Weight
**B8: Trees 25 m + distance tree patch + distance perch + Vineyard location**	**5**	**262.6**	**0**	**0.260**
B12: Null	1	263.5	0.86	0.169
B11: Vineyard location	2	263.6	0.96	0.161
B7: Box treatment + trees 25 m + distance tree patch + distance perch + Vineyard location	7	264.8	2.20	0.086
B10: Trees 25 m + distance tree patch + Vineyard location	4	265.1	2.46	0.076
B1: Box treatment × trees 25 m + box treatment × distance tree patch + distance perch + Vineyard location	11	265.2	2.61	0.071
B2: Box treatment × trees 25 m + box treatment × distance tree patch + Vineyard location	10	265.4	2.76	0.065
B3: Box treatment × distance tree patch + Trees 25 m + distance perch + Vineyard location	9	266.3	3.71	0.041
B4: Box treatment × distance tree patch + Trees 25 m + Vineyard location	8	267.0	4.44	0.028
B9: Box treatment + trees 25 m + distance tree patch + Vineyard location	6	267.8	5.21	0.019
B5: Box treatment × trees 25 m + distance tree patch + distance perch + Vineyard location	9	267.8	5.22	0.019
B6: box treatment × trees 25 m + distance tree patch + Vineyard location	8	270.8	8.22	0.004

*Note:* Table depicts binomial GLM model coefficients, parameters (*k*), AICc score, ΔAICc, and model weights. Bluebird box use is the response variable for all models. B8 (bolded) is the model with the most weight, but the null and vineyard‐only models fall within 2 ΔAICc, indicating limited support for B8 relative to competing models.

To assess whether experimentally enhanced public information boosted nest box selection especially for first‐time breeders, we compared the proportion of SYs and ASYs nesting in experimental vineyards. SY females nested in experimental vineyards at a slightly higher rate (21/36 = 58%) than ASY females (12/25 = 48%), but this difference was not significant (*χ* ^2^ = 0.29; df = 1; 95% CI [−0.14, 1]). For males, nest box selection in experimental vineyards did not differ significantly between SY (15/26 = 58%) and ASY (20/34 = 59%) individuals (*χ* ^2^ = < 0.01; df = 1; 95% CI [−0.23, 1]).

### 4.3 Reproductive Success

The top model indicated that variation in clutch size was driven primarily by clutch initiation date (Table S2). For clutches initiated later in the season, the odds of laying a larger clutch were 56% lower compared to clutches initiated earlier in the season (Table S1). Model selection for fledging success revealed a similar breeding timing pattern (Table S3). Clutches initiated later in the season had approximately 68% higher odds of nest failure in the top model (Table S1). Contrary to our prediction, female age and local habitat features were not reflected as deterministic factors in either analysis. Box treatment and vineyard location similarly had no effect on reproductive outcomes.

## 5 Discussion

Prospecting Western Bluebirds demonstrated a strong response to public information indicative of reproductive activity and breeding success. Prospecting activity increased dramatically at experimental vineyards and in particular at treatment boxes directly broadcasting cues (Figure 2). Although fewer cameras were deployed in control vineyards, nest box density was similar across all sites, and analyses were summarized per nest box, so this imbalance is unlikely to have influenced results. Control boxes were expected to receive little prospecting immediately after installation because they lacked playback cues that would have alerted birds to their newly established presence. On experimental vineyards, the immediate attraction we observed was also mediated by local habitat features. Prospecting decreased significantly with increasing distance to the nearest tree patch (Figure 3A). Moreover, the interaction between box treatment and nearby tree cover demonstrated lower prospecting rates at treatment boxes with a greater density of tree cover within close range of the nest box (Figure 4), indicating a potential trade‐off assessment of the costs and benefits of available woody cover. These findings suggest that while public information effectively alerted and initially attracted resident bluebirds to available nesting sites, it did not entirely override their assessment of vegetation structure, even for boxes directly broadcasting cues.

Additionally, the dramatic increase in prospecting triggered by simulated public information in 2023 did not lead to a clear increase in experimental nest box use in 2024. Rates of nest box use were similar across all treatments (Figure 5), with habitat‐based models outperforming treatment‐based models (Table 2). Notably, habitat features that predicted prospecting intensity also predicted settlement in one of the plausible box use models (Figure 3B). Specifically, we observed a preference for nest boxes with a lower percentage of nearby tree cover, and while not significant, the probability of box use trended negatively with increasing distance to tree patch. The reoccurrence of these trends in nest box settlement suggests that Western Bluebirds may give greater weight to personal information gathered through direct habitat sampling over public information when making final settlement decisions. However, our playback experiment was limited to a three‐day window, and it remains possible that a longer sustained period of playback may have had a stronger influence on box occupancy in the subsequent year. Moreover, it is possible that the high nest‐box densities utilized in our study (see Methods) may have resulted in slightly lower box occupancy rates across all experimental treatments. Nevertheless, occupancy rates were sufficient to detect habitat‐based patterns across both years, suggesting bluebirds may prioritize personal evaluations of broader ecological contexts when selecting nest sites.

Indeed, bluebirds prospected and showed evidence of selection for boxes balancing the benefits of available tree patches against the costs of nesting near dense natural habitat. Preference for nearby tree patches may reflect a need for shade access, which can be critical during drought conditions and heatwaves (Mooij et al. 2002; Albright et al. 2010). Higher temperatures and heat exposure have been linked to decreased nestling quality and reproductive success (Riggio et al. 2023) and reduced nestling provisioning (Tapper et al. 2020; Karp et al. 2026) in common Napa Valley nest box occupants, including Western Bluebirds and Tree Swallows. Consistent with their perch‐foraging strategy (Herlugson 1983), bluebirds also selected nest boxes closer to tall perches (≥ 3 m), which may provide vantage points for predator detection and shaded foraging opportunities that reduce thermal and energetic stress during periods of high breeding activity. However, their avoidance of nest boxes with higher surrounding tree densities may be a response to increased nest depredation risk with proximity to natural habitat; our footage recorded several instances of predators responding to playback, including Cooper's Hawks ( *Accipiter cooperii* ), Common Ravens ( *Corvus corax* ), and chipmunks, all of which are common occupants of wooded habitat and will opportunistically rob bird nests of eggs or young (Liebezeit and George 2002). Critically, the habitat preferences that we detected align with patterns observed in a broader analysis across the study region (Martinico et al. unpublished data) and are also consistent with prior descriptions of Western Bluebirds selecting edge habitat with exposed perches (Herlugson 1983).

Because Western Bluebirds retained selection for specific habitat features while prospecting and making settlement decisions, public information likely did not function as an ecological trap as it has in previous playback experiments used to lure migratory species, such as Black‐throated Blue Warblers ( *Setophaga caerulescens* ) (Betts et al. 2008) and Bobolinks ( *Dolichonyx oryzivorus* ) (Nocera et al. 2006), into subpar habitat. Resident populations that are not constrained by time and energy costs associated with migration, and have time to directly sample the environment, may rely less on social cues to expedite breeding site selection. Year‐round residence also provides a temporal buffer against migratory competitors. Western Bluebirds on our study plots initiated clutches earlier than their main box competitor, Tree Swallows, which migrate into the Central Valley from further south (Knight et al. 2018). While clutch overlap existed later in the breeding season, Western Bluebirds selected boxes in late March (ascertained from boxes already in use when checks began) and April without an abundance of competition from swallows. This low competitive pressure may have reduced the relevance of public information from the previous breeding season and allowed birds to rely on direct habitat sampling when choosing a box. Future testing in regions with more intense early‐season competition, or in contexts of increased breeding overlap due to climate‐driven shifts in phenology, may help determine whether public information becomes more influential under higher competitive pressure.

The reduced reliance on public information in our study population persisted regardless of age‐related experience; first‐time breeders did not weigh public information more heavily than older individuals during box selection. Although natal dispersers in some species exhibit greater sensitivity to public information when selecting breeding sites (Nocera et al. 2006, 2009), much of this evidence comes from migratory systems where young birds can use social cues to offset the time and energy constraints of arrival (Ahlering et al. 2010; Valente et al. 2021). In contrast, resident juveniles may face less pressure to make rapid settlement decisions. Indeed, in our study, SY females did not initiate clutches later than ASY females on either control or experimental vineyards. The lack of age‐related lag suggests that a prolonged pre‐breeding sampling period allows first‐time breeders to achieve a level of site‐familiarity similar to that of older individuals, diminishing the information gap observed between age classes in migratory systems.

Consistent with the age‐related parity in site assessment, breeder age had no discernable effect on clutch size or fledging success, contrasting our initial prediction. Had SY individuals relied on simulated social cues that were agnostic to actual nest site quality, we would expect lower reproductive success in treatment boxes where public information mismatched habitat quality. However, we instead found that reproductive success was independent of both breeder age and local habitat characteristics. Although some evidence pointed to habitat features influencing box selection, the range of habitat variation among occupied boxes may not have been large enough to generate measurable differences in reproductive success, considering their proximity to each other within each plot. Parental compensation, where adults increase effort to mitigate the costs of subpar site selection (as observed in adult Tree Swallows increasing provisioning rate to adjust for artificially enlarged broods, even when experimentally immunochallenged (Ardia 2005)), may have also buffered costs of suboptimal nest box selection. The driving factor in declining clutch size and fledging success was later clutch initiation date, consistent with general expected trends for Western Bluebirds (Keyser et al. 2004) and other songbird species (Bowman and Woolfenden 2001; Dillon and Conway 2015). Declining prey abundance, intensified heterospecific competition, and heightened predation risk later in the breeding cycle may all play roles in reduced clutch size and nest success (Bowman and Woolfenden 2001), as may hotter temperatures later in the breeding season (Karp et al. 2026).

## 6 Conclusions

For a resident population of Western Bluebirds, we found that public information acts as a powerful recruitment signal that operates in conjunction with, rather than in lieu of, structural habitat assessment. While the use of playback simulating conspecific and reproductive presence functioned to advertise newly available breeding sites, its efficacy in promoting settlement was eclipsed by preference for nest boxes in proximity to low density woody vegetation patches. This suggests that resident bluebirds may use public information as a filter to expedite the discovery of sites that can then be vetted via direct sampling. Although we observed no difference in clutch initiation timing between control and experimental vineyards, our post‐breeding design meant boxes were available for several months prior to the breeding season, which likely allowed time for resident birds to discover sites even in the absence of playback. Future pre‐breeding designs—deploying boxes and playback directly prior to the start of the breeding season—would better isolate the response time to sites with and without supplied public information and reduce the likelihood that settlement patterns are obscured by the loss of individuals over the winter months.

Integrating these behavioral insights with habitat‐based management can further improve conservation strategies aimed at attracting insect‐eating birds to agricultural landscapes. In particular, breaking up homogenous monocultures with patches of woody vegetation may incentivize box selection, in alignment with evidence that semi‐natural and non‐crop woody habitat increases avian abundance, biodiversity, and pest control services in agricultural systems (Heath et al. 2017; Kremen 2020; Garcia et al. 2023). Strategically applied playback to simulate attractive social cues and advertise higher quality breeding sites may provide more reliable nesting opportunities for birds in working landscapes.

## Author Contributions

**Fatime W. Jomaa:** conceptualization (supporting), data curation (lead), formal analysis (lead), funding acquisition (supporting), investigation (lead), methodology (equal), validation (lead), visualization (lead), writing – original draft (lead), writing – review and editing (lead). **Cody Pham:** data curation (equal), formal analysis (supporting), writing – review and editing (supporting). **Eleanor K. MacDonald:** data curation (equal), writing – review and editing (supporting). **Daniel S. Karp:** conceptualization (equal), funding acquisition (equal), methodology (equal), writing – review and editing (supporting). **Matthew D. Johnson:** conceptualization (equal), funding acquisition (equal), methodology (equal), project administration (lead), supervision (lead), writing – review and editing (supporting).

## Funding

This work was supported by Agricultural Research Institute, California State University (2023‐70440‐40177) and Humboldt Area Foundation (N4895, N4954).

## Disclosure

Statement on Inclusion: Our study utilized privately owned agricultural land across the study region. All participating vineyard owners were engaged early in the study design process. This partnership aimed to co‐produce strategies for improving the utilization of agricultural landscapes by native wildlife, with ongoing communication to share outcomes with stakeholders.

## Conflicts of Interest

The authors declare no conflicts of interest.

## Supporting information


**Table S1:** Model coefficient tables for top models. Tables report coefficients, standard errors, Z values, and 95% confidence intervals.
**Table S2:** Clutch size candidate model set.
**Table S3:** Fledge success candidate model set.

## Data Availability

Data available from the Zenodo Repository: https://doi.org/10.5281/zenodo.19428948.

## References

[ece374210-bib-0001] 1/2″ Conduit/Rebar Pole Mounting System . 2012. https://michiganbluebirds.org/images/stories/easygallery/downloads/conduit%20rebar%20mounting.pdf.

[ece374210-bib-0002] Ahlering, M. A. , D. Arlt , M. G. Betts , R. J. Fletcher Jr. , J. J. Nocera , and M. P. Ward . 2010. “Research Needs and Recommendations for the Use of Conspecific‐Attraction Methods in the Conservation of Migratory Songbirds.” Condor 112, no. 2: 252–264. 10.1525/cond.2010.090239.

[ece374210-bib-0003] Akaike, H. 2025. “Akaike's Information Criterion.” In International Encyclopedia of Statistical Science, 41–42. Springer. 10.1007/978-3-662-69359-9_14.

[ece374210-bib-0004] Albright, T. P. , A. M. Pidgeon , C. D. Rittenhouse , et al. 2010. “Combined Effects of Heat Waves and Droughts on Avian Communities Across the Conterminous United States.” Ecosphere 1, no. 5: 1–22. 10.1890/ES10-00057.1.

[ece374210-bib-0005] Ardia, D. R. 2005. “Individual Quality Mediates Trade‐Offs Between Reproductive Effort and Immune Function in Tree Swallows.” Journal of Animal Ecology 74, no. 3: 517–524. 10.1111/j.1365-2656.2005.00950.x.

[ece374210-bib-0006] Bartoń, K. 2025. “MuMIn: Multi‐Model Inference.” R package version 1.48. 11.

[ece374210-bib-0007] Bates, D. , M. Mächler , B. Bolker , and S. Walker . 2015. “Fitting Linear Mixed‐Effects Models Using lme4.” Journal of Statistical Software 67: 1–48. 10.18637/jss.v067.i01.

[ece374210-bib-0008] Betts, M. G. , A. S. Hadley , N. Rodenhouse , and J. J. Nocera . 2008. “Social Information Trumps Vegetation Structure in Breeding‐Site Selection by a Migrant Songbird.” Proceedings of the Royal Society B: Biological Sciences 275, no. 1648: 2257–2263. 10.1098/rspb.2008.0217. PMC2603235 18559326

[ece374210-bib-0009] Boulinier, T. , E. Danchin , J. Y. Monnat , C. Doutrelant , and B. Cadiou . 1996. “Timing of Prospecting and the Value of Information in a Colonial Breeding Bird.” Journal of Avian Biology 27: 252–256. 10.2307/3677230.

[ece374210-bib-0010] Bouvier, J. C. , T. Delattre , T. Boivin , R. Musseau , C. Thomas , and C. Lavigne . 2022. “Great Tits Nesting in Apple Orchards Preferentially Forage in Organic but Not Conventional Orchards and in Hedgerows.” Agriculture, Ecosystems & Environment 337: 108074. 10.1016/j.agee.2022.108074.

[ece374210-bib-0011] Bowman, R. , and G. E. Woolfenden . 2001. “Nest Success and the Timing of Nest Failure of Florida Scrub‐Jays in Suburban and Wildland Habitats.” In Avian Ecology and Conservation in an Urbanizing World, 383–402. Springer US. 10.1007/978-1-4615-1531-9_18.

[ece374210-bib-0012] Brandl, H. B. , S. C. Griffith , T. Laaksonen , and W. Schuett . 2019. “Begging Calls Provide Social Cues for Prospecting Conspecifics in the Wild Zebra Finch ( *Taeniopygia guttata* ).” Auk: Ornithological Advances 136, no. 2: ukz007. 10.1093/auk/ukz007.

[ece374210-bib-0013] Brooks, M. E. , K. Kristensen , K. J. Van Benthem , et al. 2017. “glmmTMB Balances Speed and Flexibility Among Packages for Zero‐Inflated Generalized Linear Mixed Modeling.” 10.32614/RJ-2017-066.

[ece374210-bib-0014] Brown, J. L. , M. W. Collopy , and J. A. Smallwood . 2014. “Habitat Fragmentation Reduces Occupancy of Nest Boxes by an Open‐Country Raptor.” Bird Conservation International 24, no. 3: 364–378. 10.1017/S0959270913000415.

[ece374210-bib-0015] Charnov, E. L. , and J. R. Krebs . 1974. “On Clutch‐Size and Fitness.” Ibis 116, no. 2: 217–219. 10.1111/j.1474-919X.1974.tb00241.x.

[ece374210-bib-0016] Christensen, R. H. B. 2019. “Ordinal—Regression Models for Ordinal Data.” R Package Version, 10(2019), 54.

[ece374210-bib-0017] Cody, M. L. 1981. “Habitat Selection in Birds: The Roles of Vegetation Structure, Competitors, and Productivity.” Bioscience 31, no. 2: 107–113. 10.2307/1308252.

[ece374210-bib-0018] Crampton, L. H. , W. S. Longland , D. D. Murphy , and J. S. Sedinger . 2011. “Food Abundance Determines Distribution and Density of a Frugivorous Bird Across Seasons.” Oikos 120, no. 1: 65–76. 10.1111/j.1600-0706.2010.18624.x.

[ece374210-bib-0019] Danchin, É. , L. A. Giraldeau , T. J. Valone , and R. H. Wagner . 2004. “Public Information: From Nosy Neighbors to Cultural Evolution.” Science 305, no. 5683: 487–491. 10.1126/science.1098254. 15273386

[ece374210-bib-0020] Dillon, K. G. , and C. J. Conway . 2015. “Elevational Gradient in Clutch Size of Red‐Faced Warblers.” Journal of Field Ornithology 86, no. 2: 163–172. 10.1111/jofo.12099.

[ece374210-bib-0021] Doligez, B. , T. Pärt , and E. Danchin . 2004. “Prospecting in the Collared Flycatcher: Gathering Public Information for Future Breeding Habitat Selection?” Animal Behaviour 67, no. 3: 457–466. 10.1016/j.anbehav.2003.03.010.

[ece374210-bib-0022] Duckworth, R. A. 2008. “Adaptive Dispersal Strategies and the Dynamics of a Range Expansion.” American Naturalist 172, no. S1: S4–S17. 10.1086/588289. 18554143

[ece374210-bib-0023] Dunlop, C. L. , H. Blokpoel , and S. Jarvie . 1991. “Nesting Rafts as a Management Tool for a Declining Common Tern ( *Sterna hirundo* ) Colony.” Colonial Waterbirds 14: 116–120. 10.2307/1521499.

[ece374210-bib-0024] Gaiser, T. 2025. Capstone California. California Wine Institute.

[ece374210-bib-0025] Garcia, K. , E. M. Olimpi , L. M'Gonigle , et al. 2023. “Semi‐Natural Habitats on Organic Strawberry Farms and in Surrounding Landscapes Promote Bird Biodiversity and Pest Control Potential.” Agriculture, Ecosystems & Environment 347: 108353. 10.1016/j.agee.2023.108353.

[ece374210-bib-0026] Hartig, F. 2024. “DHARMa: Residual Diagnostics for Hierarchical (Multi‐Level/Mixed) Regression Models.” R Package Version 0.4.7.2024.

[ece374210-bib-0027] Heath, S. K. , C. U. Soykan , K. L. Velas , R. Kelsey , and S. M. Kross . 2017. “A Bustle in the Hedgerow: Woody Field Margins Boost on Farm Avian Diversity and Abundance in an Intensive Agricultural Landscape.” Biological Conservation 212: 153–161. 10.1016/j.biocon.2017.05.031.

[ece374210-bib-0028] Herlugson, C. J. 1983. “Hover‐Foraging by Western Bluebirds.” Murrelet 64, no. 2: 58–60. 10.2307/3534694.

[ece374210-bib-0029] Jedlicka, J. A. , A. T. E. Vo , and R. P. Almeida . 2017. “Molecular Scatology and High‐Throughput Sequencing Reveal Predominately Herbivorous Insects in the Diets of Adult and Nestling Western Bluebirds ( *Sialia mexicana* ) in California Vineyards.” Auk: Ornithological Advances 134, no. 1: 116–127. 10.1642/AUK-16-103.1.

[ece374210-bib-0030] Jeffries, D. S. , and D. H. Brunton . 2001. “Attracting Endangered Species to ‘Safe’ Habitats: Responses of Fairy Terns to Decoys.” Animal Conservation 4, no. 4: 301–305. 10.1017/S1367943001001354.

[ece374210-bib-0031] Johnson, M. D. , and E. M. Wood . 2018. “Habitat Ecology.” In Ornithology: Foundation, Analysis, and Application, edited by M. L. Morrison , A. D. Rodewald , G. Voelker , J. F. Prather , and M. R. Colón , 579–612. Johns Hopkins Press. 10.56021/9781421424712.

[ece374210-bib-0033] Karp, D. S. , J. A. Smith , C. Pham , et al. 2026. Avian Behavioral Thermoregulation Carries Reproductive Opportunity Costs During Heat Waves in Agriculture [Unpublished manuscript]. Department of Wildlife, Fish and Conservation Biology, University of California, Davis.

[ece374210-bib-0034] Kelly, J. K. , and K. A. Schmidt . 2017. “Fledgling Calls Are a Source of Social Information for Conspecific, but Not Heterospecific, Songbird Territory Selection.” Ecosphere 8, no. 2: e01512. 10.1002/ecs2.1512.

[ece374210-bib-0035] Keyser, A. J. , M. T. Keyser , and D. E. Promislow . 2004. “Life‐History Variation and Demography in Western Bluebirds ( *Sialia mexicana* ) in Oregon.” Auk 121, no. 1: 118–133. 10.1093/auk/121.1.118.

[ece374210-bib-0036] Knight, S. M. , D. W. Bradley , R. G. Clark , et al. 2018. “Constructing and Evaluating a Continent‐Wide Migratory Songbird Network Across the Annual Cycle.” Ecological Monographs 88, no. 3: 445–460. 10.1002/ecm.1298.

[ece374210-bib-0037] Kraaijeveld, K. , and J. L. Dickinson . 2001. “Family‐Based Winter Territoriality in Western Bluebirds, *Sialia mexicana* : The Structure and Dynamics of Winter Groups.” Animal Behaviour 61, no. 1: 109–117. 10.1006/anbe.2000.1591. 11170701

[ece374210-bib-0038] Kremen, C. 2020. “Ecological Intensification and Diversification Approaches to Maintain Biodiversity, Ecosystem Services and Food Production in a Changing World.” Emerging Topics in Life Sciences 4, no. 2: 229–240. 10.1042/ETLS20190205. 32886114 PMC7487174

[ece374210-bib-0039] Kross, S. M. , and R. A. Baldwin . 2016. “Gopherbusters? A Review of the Candidacy of Barn Owls as the Ultimate Natural Pest Control Option.” In Proceedings of the Vertebrate Pest Conference, vol. 27, No. 27. eScholarship Publishing. 10.5070/V427110691.

[ece374210-bib-0040] Liebezeit, J. R. , and T. L. George . 2002. “Nest Predators, Nest‐Site Selection, and Nesting Success of the Dusky Flycatcher in a Managed Ponderosa Pine Forest.” Condor 104, no. 3: 507–517. 10.1093/condor/104.3.507.

[ece374210-bib-0041] Lüdecke, D. , M. S. Ben‐Shachar , I. Patil , P. Waggoner , and D. Makowski . 2021. “Performance: An R Package for Assessment, Comparison and Testing of Statistical Models.” Journal of Open Source Software 6, no. 60: 3139. 10.21105/joss.03139.

[ece374210-bib-0042] Mooij, W. M. , R. E. Bennetts , W. M. Kitchens , and D. L. DeAngelis . 2002. “Exploring the Effect of Drought Extent and Interval on the Florida Snail Kite: Interplay Between Spatial and Temporal Scales.” Ecological Modelling 149, no. 1–2: 25–39. 10.1016/S0304-3800(01)00512-9.

[ece374210-bib-0043] Mummert, D. P. , L. Baines , and W. D. Tietje . 2002. “Cavity‐Nesting Bird Use of Nest Boxes in Vineyards of Central‐Coast California^1^ .” In Proceedings of the Fifth Symposium on Oak Woodlands: Oaks in California's Changing Landscape, October 22–25, 2001, San Diego, California, vol. 184, 335. US Department of Agriculture, Forest Service, Pacific Southwest Research Station.

[ece374210-bib-0044] Napa County . 2010. “Napa County Voluntary Oak Woodland Management Plan.” Napa County, Napa, California, USA.

[ece374210-bib-0045] Navara, K. J. , and E. M. Anderson . 2011. “Eastern Bluebirds Choose Nest Boxes Based on Box Orientation.” Southeastern Naturalist 10, no. 4: 713–720. 10.1656/058.010.0410.

[ece374210-bib-0046] Nocera, J. J. , G. J. Forbes , and L. A. Giraldeau . 2006. “Inadvertent Social Information in Breeding Site Selection of Natal Dispersing Birds.” Proceedings of the Royal Society B: Biological Sciences 273, no. 1584: 349–355. 10.1098/rspb.2005.3318. PMC1560037 16543178

[ece374210-bib-0047] Nocera, J. J. , G. J. Forbes , and L. A. Giraldeau . 2009. “Aggregations From Using Inadvertent Social Information: A Form of Ideal Habitat Selection.” Ecography 32, no. 1: 143–152. 10.1111/j.1600-0587.2008.05614.x.

[ece374210-bib-0048] North American Bird Conservation Initiative [NABCI], U. S. Committee . 2025. The State of the Birds 2025. Cornell Lab of Ornithology.

[ece374210-bib-0049] Pärt, T. , D. Arlt , B. Doligez , M. Low , and A. Qvarnström . 2011. “Prospectors Combine Social and Environmental Information to Improve Habitat Selection and Breeding Success in the Subsequent Year.” Journal of Animal Ecology 80, no. 6: 1227–1235. 10.1111/j.1365-2656.2011.01854.x. 21569028

[ece374210-bib-0050] Pärt, T. , and B. Doligez . 2003. “Gathering Public Information for Habitat Selection: Prospecting Birds Cue on Parental Activity.” Proceedings of the Royal Society of London. Series B: Biological Sciences 270, no. 1526: 1809–1813. 10.1098/rspb.2003.2419. PMC1691451 12964983

[ece374210-bib-0051] Podolsky, R. , and S. W. Kress . 1992. “Attraction of the Endangered Dark‐Rumped Petrel to Recorded Vocalizations in the Galapagos Islands.” Condor 94, no. 2: 448–453. 10.2307/1369217.

[ece374210-bib-0052] Purcell, K. L. 2011. “Long‐Term Avian Research at the San Joaquin Experimental Range: Recommendations for Monitoring and Managing Oak Woodlands.” Forest Ecology and Management 262, no. 1: 12–19. 10.1016/j.foreco.2010.07.039.

[ece374210-bib-0053] R Core Team . 2024. R: A Language and Environment for Statistical Computing. R Foundation for Statistical Computing.

[ece374210-bib-0054] Reed, J. M. , T. Boulinier , E. Danchin , and L. W. Oring . 1999. “Informed Dispersal: Prospecting by Birds for Breeding Sites.” In Current Ornithology, 189–259. Springer US.

[ece374210-bib-0055] Riggio, J. , A. Engilis Jr. , H. Cook , E. De Greef , D. S. Karp , and M. L. Truan . 2023. “Long‐Term Monitoring Reveals the Impact of Changing Climate and Habitat on the Fitness of Cavity‐Nesting Songbirds.” Biological Conservation 278: 109885. 10.1016/j.biocon.2022.109885.

[ece374210-bib-0056] Scrucca, L. 2018. “Dispmod: Modelling Dispersion in GLM.” R package version 1.2.

[ece374210-bib-0057] Smith, M. G. , Ç. Akçay , D. Shizuka , C. A. Stern , and J. L. Dickinson . 2020. “Extraterritorial Visits in a Cooperatively Breeding Songbird Are Consistent With Multiple Functions.” Animal Behaviour 170: 119–132. 10.1016/j.anbehav.2020.10.012.

[ece374210-bib-0058] Tapper, S. , J. J. Nocera , and G. Burness . 2020. “Experimental Evidence That Hyperthermia Limits Offspring Provisioning in a Temperate‐Breeding Bird.” Royal Society Open Science 7, no. 10: 201589. 10.1098/rsos.201589. 33204485 PMC7657879

[ece374210-bib-0059] Thornton, M. M. , R. Shrestha , Y. Wei , P. E. Thornton , and S.‐C. Kao . 2020. “Daymet: Daily Surface Weather Data on a 1‐km Grid for North America, Version 4.” ORNL DAAC, Oak Ridge, Tennessee, USA. 10.3334/ORNLDAAC/1840.

[ece374210-bib-0060] Valente, J. J. , C. L. LeGrande‐Rolls , J. W. Rivers , A. M. Tucker , R. A. Fischer , and M. G. Betts . 2021. “Conspecific Attraction for Conservation and Management of Terrestrial Breeding Birds: Current Knowledge and Future Research Directions.” Condor 123, no. 2: duab007. 10.1093/ornithapp/duab007.

[ece374210-bib-0061] Valone, T. J. , and J. J. Templeton . 2002. “Public Information for the Assessment of Quality: A Widespread Social Phenomenon.” Philosophical Transactions of the Royal Society of London. Series B: Biological Sciences 357, no. 1427: 1549–1557. 10.1098/rstb.2002.1064. 12495512 PMC1693063

[ece374210-bib-0062] Ward, M. P. , and S. Schlossberg . 2004. “Conspecific Attraction and the Conservation of Territorial Songbirds.” Conservation Biology 18, no. 2: 519–525. 10.1111/j.1523-1739.2004.00494.x.

[ece374210-bib-0063] Wendt, C. A. , and M. D. Johnson . 2017. “Multi‐Scale Analysis of Barn Owl Nest Box Selection on Napa Valley Vineyards.” Agriculture, Ecosystems & Environment 247: 75–83. 10.1016/j.agee.2017.06.023.

[ece374210-bib-0064] Williams, G. C. 1966. “Natural Selection, the Costs of Reproduction, and a Refinement of Lack's Principle.” American Naturalist 100, no. 916: 687–690. 10.1086/282461.

[ece374210-bib-0065] Winter, M. , D. H. Johnson , and J. A. Shaffer . 2005. “Variability in Vegetation Effects on Density and Nesting Success of Grassland Birds.” Journal of Wildlife Management 69, no. 1: 185–197. 10.2193/0022-541X(2005)069<0185:VIVEOD>2.0.CO;2.

