# Supplementary material for: Western Bluebirds (Sialia mexicana) Use Public Information for Prospecting but Not Ultimate Nest Site Selection
Source: Ecol Evol. 2026 Aug 18;16(8):e74210. doi: 10.1002/ece3.74210 (PMC13482770; doi:10.1002/ece3.74210)
Supplement: Supplementary file 1 [file ECE3-16-e74210-s001.docx]

**Table S1:** Model coefficient tables for top models. Tables report coefficients, standard errors, Z values, and 95% confidence intervals. Significant effects are bolded. For the zero-inflated negative binomial model of prospecting activity, results are presented separately for the count component and the zero-inflation (ZI) component. For the binomial GLM box use models, the null and vineyard location models that fall within 2 ΔAICc of the habitat model are included as well. For the ordinal logistic regression model of clutch size, threshold parameters represent the model’s internal cut‑points on an underlying continuous scale of clutch size. For example, the 3|4 threshold marks the point where the model shifts from predicting a clutch of 3 eggs to predicting 4 eggs. These values are structural components of the ordinal model and do not represent biological effects.

| Response | Variables | | Coef. | SE | Z | 95% CI |
| --- | --- | --- | --- | --- | --- | --- |
| Prospecting Activity (P6) | Count Model | Intercept | 2.15 | 0.29 | 7.42 | **1.59, 2.72** |
|  |  | Box Treatment (Near-treatment) | 1.02 | 0.34 | 3.04 | **0.36, 1.68** |
|  |  | Box Treatment (Treatment) | 2.14 | 0.31 | 6.93 | **1.53, 2.74** |
|  |  | Trees 25m | -0.33 | 0.31 | -1.06 | -0.93, 0.28 |
|  |  | Distance Tree Patch | -0.50 | 0.12 | -3.99 | **-0.74, -0.25** |
|  |  | Box Treatment (Near-treatment) * Trees 25m | -0.07 | 0.26 | 2.10 | -0.78, 0.65 |
|  |  | Box Treatment (Treatment) * Trees 25m | -1.00 | 0.36 | -0.19 | **-1.68, -0.33** |
|  |  | Vineyard Location (Valley Floor) | 0.54 | 0.34 | -2.92 | **0.04, 1.04** |
|  | ZI Model | Intercept | -0.56 | 0.42 | -1.35 | -1.38, 0.25 |
|  |  | Box Treatment (Near-treatment) | -1.91 | 0.99 | -1.94 | -3.84, 0.02 |
|  |  | Box Treatment (Treatment) | -2.97 | 1.20 | -2.47 | **-5.33, -0.61** |
| Box Use (B8) | Habitat model | Intercept | -0.51 | 0.24 | -2.09 | **-1, -0.03** |
|  |  | Trees 25m | -0.49 | 0.22 | -2.21 | **-0.94, -0.06** |
|  |  | Distance Tree Patch | -0.21 | 0.19 | -1.10 | -0.63, 0.14 |
|  |  | Distance Perch | -0.47 | 0.24 | -1.99 | **-0.98, -0.04** |
|  |  | Vineyard Location (Valley Floor) | -0.48 | 0.35 | -1.40 | -1.17, 0.19 |
| Box Use (B12) | Null model | Intercept | -0.74 | 0.15 | -5.01 | **-1.04, -0.46** |
| Box Use (B11) | Vineyard Location model | Intercept | -0.51 | 0.22 | -2.32 | **-0.95, -0.09** |
|  |  | Vineyard Location (Valley Floor) | -0.42 | -0.30 | -1.40 | -1.01, 0.17 |
| Clutch Size (C8) | Predictors | Clutch Initiation Date | -0.81 | 0.27 | -2.98 | **-1.35, -0.28** |
|  |  | Vineyard Location (Valley Floor) | -0.67 | 0.50 | -1.34 | -1.66, 0.31 |
|  | Thresholds | 3 \| 4 | -3.35 | 0.63 | -5.30 | **-4.59, -2.11** |
|  |  | 4 \| 5 | -1.21 | 0.41 | -2.96 | **-2.01, -0.41** |
|  |  | 5 \| 6 | 1.64 | 0.43 | 3.82 | **0.80, 2.48** |
| Fledging Success (F8) |  | Intercept | 1.36 | 0.22 | 6.30 | **0.95, 1.80** |
|  |  | Clutch Initiation Date | -0.52 | 0.16 | -3.28 | **-0.83, -0.21** |
|  |  | Vineyard Location (Valley Floor) | 0.02 | 0.32 | 0.07 | -0.60, 0.65 |

**Table S2:** Clutch size candidate model set. Table depicts ordinal logistic regression model coefficients, parameters (k), AICc score, ΔAICc, and model weights. Western Bluebird clutch size is the ordered response variable for all models. C8 (bolded) is the top model with no other models falling within 2 ΔAICc.

| Model | k | AICc | ΔAICc | Weight |
| --- | --- | --- | --- | --- |
| **C8: Clutch Initiation Date + Vineyard Location** | **5** | **140.2** | **0** | **0.887** |
| C7: Trees 25m + Distance Tree Patch + Distance Perch + Cutch Initiation Date + Vineyard Location | 8 | 146.6 | 6.39 | 0.036 |
| C10: Null | 3 | 146.8 | 6.56 | 0.033 |
| C9: Vineyard Location | 4 | 147.3 | 7.07 | 0.026 |
| C6: Female Age + Trees 25m + Distance Tree Patch + Distance Perch + Clutch Initiation Date + Vineyard Location | 9 | 148.9 | 8.72 | 0.011 |
| C5: Box Treatment + Trees 25m + Distance Tree Patch + Distance Perch + Clutch Initiation Date + Vineyard Location | 10 | 151.1 | 10.84 | 0.004 |
| C2: Female Age * Trees 25m + Female Age * Distance Tree Patch + Distance Perch + Clutch Initiation Date + Vineyard Location | 11 | 153.7 | 13.49 | 0.001 |
| C4: Female Age + Box Treatment + Trees 25m + Distance Tree Patch + Distance Perch + Clutch Initiation Date + Vineyard Location | 11 | 153.9 | 13.67 | 0.001 |
| C3: Female Age * Box Treatment + Trees 25m + Distance Tree Patch + Distance Perch + Clutch Initiation Date + Vineyard Location | 13 | 158.4 | 18.13 | 0 |
| C1: Female Age * Box Treatment + Female Age * Trees 25m + Female Age * Distance Tree Patch + Distance Perch + Clutch Initiation Date + Vineyard Location | 15 | 165.1 | 24.86 | 0 |

**Table S3:** Fledge success candidate model set. Table depicts ordinal logistic regression model coefficients, parameters (k), AICc score, ΔAICc, and model weights. Western Bluebird clutch size is the ordered response variable for all models. F8 (bolded) is the top model with no other models falling within 2 ΔAICc.

| Model | k | AICc | ΔAICc | Weight |
| --- | --- | --- | --- | --- |
| **F8: Clutch Initiation Date + Vineyard Location** | **3** | **68.0** | **0** | **0.887** |
| F10: Null | 1 | 70.1 | 2.09 | 0.195 |
| F7: Trees 25m + Distance Tree Patch + Distance Perch + Cutch Initiation Date + Vineyard Location | 6 | 71.1 | 3.10 | 0.118 |
| F9: Vineyard Location | 2 | 71.6 | 3.54 | 0.094 |
| F6: Female Age + Trees 25m + Distance Tree Patch + Distance Perch + Clutch Initiation Date + Vineyard Location | 7 | 75.2 | 7.21 | 0.015 |
| F5: Box Treatment + Trees 25m + Distance Tree Patch + Distance Perch + Clutch Initiation Date + Vineyard Location | 8 | 75.3 | 7.29 | 0.015 |
| F4: Female Age + Box Treatment + Trees 25m + Distance Tree Patch + Distance Perch + Clutch Initiation Date + Vineyard Location | 9 | 77.1 | 9.09 | 0.006 |
| F2: Female Age * Trees 25m + Female Age * Distance Tree Patch + Distance Perch + Clutch Initiation Date + Vineyard Location | 9 | 80.5 | 12.45 | 0.001 |
| F3: Female Age * Box Treatment + Trees 25m + Distance Tree Patch + Distance Perch + Clutch Initiation Date + Vineyard Location | 11 | 81.8 | 13.75 | 0.001 |
| F1: Female Age * Box Treatment + Female Age * Trees 25m + Female Age * Distance Tree Patch + Distance Perch + Clutch Initiation Date + Vineyard Location | 13 | 84.9 | 16.86 | 0 |
